# ‘Just because we're younger doesn't mean our opinions should be any less valued’: A qualitative study of youth perspectives on a Youth Advisory Council in a mental healthcare context

**DOI:** 10.1111/hex.13794

**Published:** 2023-06-16

**Authors:** Adrienne Young, Anthony Levitt, Sugy Kodeeswaran, Roula Markoulakis

**Affiliations:** ^1^ Sunnybrook Research Institute Toronto Ontario Canada; ^2^ York University School of Social Work Toronto Ontario Canada; ^3^ Sunnybrook Health Sciences Centre Toronto Ontario Canada; ^4^ Temerty Faculty of Medicine University of Toronto Toronto Ontario Canada; ^5^ Department of Occupational Science and Occupational Therapy, Faculty of Medicine University of Toronto Toronto Ontario Canada

**Keywords:** lived experience, mental health, navigation, Youth Advisory Council, youth engagement

## Abstract

**Introduction:**

Recognition of the importance of youth engagement in youth mental health and/or addiction (MHA) service design and delivery is increasing. Youth Advisory Councils embedded in MHA organizations represent one strategy that allows youth to be involved in MHA at the individual, organization and systemic levels. This level of involvement can facilitate positive outcomes for both the youth and the organization. As these councils become more common, it is important that organizations are prepared to partner with the participating youth. This study uses a descriptive qualitative approach to understand the motivations and expectations of youth with lived experience with MHA concerns who were beginning to work on a Youth Advisory Council in an MHA setting in the Greater Toronto Area.

**Methods:**

Semistructured interviews were conducted with each youth, ages 16–26, on the advisory council (*N* = 8) to understand their motivations, expectations and goals coming into the work. Interviews were transcribed verbatim and analysed using a reflexive thematic analysis.

**Results:**

Analysis resulted in five overarching themes: providing opportunities for youth learning and growth, platforming youth, empowering youth, embracing youth leadership and promoting youth‐driven change. The findings illustrate that these youth came into the Youth Advisory Council motivated to create positive change in the mental health system, take on leadership roles and had high expectations for organizational support. Our analyses provide insight for organizations planning and implementing Youth Advisory Councils in the MHA sector with the goal of best supporting youth in driving positive change across the system.

**Conclusion:**

Youth want to be provided authentic opportunities for their engagement to make a difference. MHA organizations must embrace youth leadership and move towards listening to youth experience and acting on youth recommendations to enhance service design and implementation to improve access and to better meet the needs of youth utilizing these services.

**Patient or Public Contributions:**

This study incorporated service users, including youth ages 16–26 with lived experience of MHA concerns who served on the Youth Advisory Council at the Family Navigation Project, Sunnybrook. Youth Advisory Council members contributed to two relevant research activities: (1) youth reviewed the draft interview guide before data collection, and their feedback was prioritized in the final version and (2) youth contributed to knowledge translation through contributing to academic conference presentations.

## 1 INTRODUCTION

Mental health and/or addiction (MHA) issues impact an estimated 1.2 million Canadian children and youth.^1^ In fact, youth mental health concerns make up the largest portion of the global health‐related burden for youth.^2^ The youth mental health crisis was exacerbated by the coronavirus disease 2019 (COVID‐19) pandemic, with youth around the world reporting decreases in well‐being and mental health deterioration.^3^, ^4^, ^5^, ^6^ Still, fewer than 20% of Canadian youth receive appropriate MHA treatment.^1^ These figures are particularly concerning as early intervention is critical to reducing the burden of illness across the lifespan.^7^ Youth engagement at the organizational level is an emerging strategy to combat the youth mental health crisis.^8^, ^9^, ^10^, ^11^, ^12^

Youth engagement has been described as the process of participation of youth at the community and organization level that is made meaningful through intentional consideration of youth strengths, interests and developmental needs.^13^ Meaningful youth engagement draws on Article 12 of the United Nations Convention of the Rights of the Child, which stipulates that decision‐makers must solicit youth input in matters that impact them.^14^ Positive youth development (PYD) has been posited as a framework for meaningful youth engagement,^15^ as it takes a strength‐based view towards youth and stresses the importance of creating opportunities for youth to gain skills, build relationships, become leaders and to thrive in adulthood.^16^, ^17^

There is increasing evidence of the benefits of youth engagement within healthcare organizations for both the youth and the organization through providing opportunities for participating youth to grow personally and professionally^15^, ^18^, ^19^, ^20^ and encouraging the development of community‐specific and contextually relevant organizational programming.^9^, ^21^, ^22^ Youth Advisory Councils (YACs), sometimes called Youth Advisory Boards, Committees, Coalitions or Groups, represent one way that healthcare organizations can structure youth engagement to lift youth voices within healthcare systems to identify youth priorities for the MHA sector.^18^, ^23^, ^24^, ^25^, ^26^

YACs have been increasing in popularity in the healthcare field over the last two decades.^27^ YACs help organizations find ways to reduce barriers and create pathways to more meaningful client engagement.^25^, ^27^ Prior research on youth engagement in MHA has evaluated youth personal and professional growth,^28^, ^29^ experiences and required work of adult facilitators and organizations^18^, ^30^ and outcomes of youth engagement such as the development of youth‐friendly resources.^10^ Less is known about what motivates youth to get involved at the advisory level in MHA, and what expectations youth bring into the work. Our research objectives are to explore (1) why youth want to be involved in advisory roles in MHA and (2) what they expect from the experience through analysis of interviews with eight youth beginning their work on a YAC in the MHA sector. This understanding may help MHA organizations as they develop and grow their youth engagement strategies.

## 2 STUDY CONTEXT

The Family Navigation Project (FNP) at Sunnybrook is a nonprofit organization that provides MHA navigation support for youth aged 13–26 and their families.^31^ MHA navigation programmes work through identifying pathways to and through the multitudes of MHA treatment options to help individuals and their families find appropriate care.^32^, ^33^, ^34^ FNP is a free service where youth or caregivers of youth can connect with a navigator to receive individualized support to find and access appropriate and timely MHA care.^31^

Based on guidance from youth with lived experience with MHA concerns, and prior evidence from FNP's research pertaining to a youth engagement framework in MHA navigation services, FNP established their first YAC in February 2022.^35^, ^36^, ^37^ The YAC was guided by PYD, encouraging opportunities for youth to gain skills to accomplish their goals, build on their established strengths and provide platforms for youth to share their insights and recommendations. The YAC meets monthly virtually via Zoom to guide the implementation of FNP's Youth Engagement Strategy^36^ through providing feedback on content areas chosen by FNP and through the development of a youth‐led project to increase youth engagement at FNP. Youth advisors are compensated for their participation and expertise. The first term of the YAC ran from February 2022 to September 2022.

## 3 METHODS

### 3.1 Study design

This qualitative study used a descriptive qualitative approach to explore the experiences of youth as they joined the FNP YAC. A descriptive qualitative approach encourages researchers to remain close to the qualitative data source to comprehensively describe events in the words of event participants.^38^ Study methods and results are presented in accordance with the COREQ (COnsolidated criteria for REporting Qualitative research) checklist.^39^ This study is part of a larger investigation into the facilitators and barriers to youth engagement through YACs in MHA system navigation. All study protocols were approved by Sunnybrook Health Sciences Research Centre Research Ethics Board (#5476).

### 3.2 Recruitment for YAC

At the time of the launch, FNP was working with four Youth Engagement Partners who informed the development of the YAC and joined as the first members. Youth Engagement Partners worked with FNPs in their first year to guide a participatory research study to develop a youth engagement framework.^37^ FNP conducted outreach to attract additional youth through MHA agencies in the Greater Toronto Area, focusing on those who served equity‐deserving groups. Applicants completed a written application and virtual interview to share their ideas about improving the MHA system, the importance of diversity, equity, inclusion and belonging, as well as their strengths for the role. Including the four former Youth Engagement Partners, a total of eight youths aged 16–26, from diverse backgrounds, with lived experience with MHA concerns and who lived in the Greater Toronto Area, were selected out of 55 applicants for the first term of the FNP YAC. Applicants with a strong commitment to equity and a desire to create positive change in the MHA system were chosen based on recommendations from the Youth Engagement Partners. The age range was set to 16–26, as at age 16, the youth could consent for themselves, and 26 is the age cap for FNP services. More demographic information about the members of the inaugural YAC is provided in Table 1.

**Table 1:** Demographic information of participants.

	All participants (*n* = 8)
Age	Average: 21.5
	Range: 16–26
Race/ethnicity	
South Asian	25%
White	25%
Mixed race (Black, White)	12.5%
Mixed race (Indigenous, White)	12.5%
Indo‐Caribbean	12.5%
Southeast Asian	12.5%
Living with a disability	75%
2SLGBTQI+	50%
Student	62.5%
Previous professional experience	
MHA organization	37.5%
Youth engagement	50%

Abbreviations: 2SLGBTQI+, Two Spirit, Lesbian, Gay, Bisexual, Trans, Queer, Questioning, Intersex and more; MHA, mental health and/or addiction.

### 3.3 Data collection

Each youth advisor (*n* = 8) completed a single semistructured interview via Zoom. The interviewer (A. Y.), who served as the organization liaison for the YAC, has a masters in social work, identifies as female, mixed race (Indigenous, White) and was 28 years old at the time of the interviews. As organization liaison, she attended all YAC meetings, bridged communication between the organization and the YAC, provided administrative assistance and was available for support as requested by YAC members. The interviewer had a previous working relationship with half of the group (former Youth Engagement Partners, *n* = 4) at the time of the interviews.

Interviews were conducted at the start of the first term of the YAC with some participants having participated in the YAC orientation. Interviews were audio recorded and transcribed verbatim. Interview guides were developed by the organization liaison in partnership with youth and explored youth advisors' motivation to join the FNP YAC, their expectations coming into the work and their goals for the upcoming term. Sample questions can be seen in Table 2. Interviews lasted on average 38 min. The interviewer completed memos after each interview to facilitate a deeper exploration of data.^40^

**Table 2:** Sample interview questions.

Interview topic	Sample interview questions
Motivation	What interested you about the YAC that made you want to get involved? What excites you about this opportunity?
Expectations	What are your expectations of FNP for this work? What are your expectations of your fellow YAC members for this work? How do you think organizations and the adults within them should prepare to work with youth for projects like these?
Goals	Do you have a personal goal for yourself on this project? Does this work support your larger career or life goals? Are there things that you'd like to learn about or be trained in to better perform your role as a YAC member?

Abbreviations: FNP, Family Navigation Project; YAC, Youth Advisory Council.

### 3.4 Data analysis

Data were analysed using reflexive thematic analysis.^41^, ^42^ Consistent with Braun and Clarke,^41^ the analysis included six phases. Two coders (A. Y. and R. M.) familiarized themselves with the data and coded independently in the first phase using MAXQDA. The codes were refined and reorganized through reflexive discussion between coders (A. Y. and R. M.), including resolving any disagreement, in virtual meetings until saturation was reached. Once all the transcripts had been coded, both coders (A. Y. and R. M.) reviewed the memo and codes together to search for themes, and sorted codes into corresponding overarching and subthemes. Coders (A. Y. and R. M.) sorted the 86 total codes into 17 subthemes. The 17 subthemes were then sorted into five overarching themes. Finally, coders (A. Y. and R. M.) defined and named the overarching themes.

## 4 RESULTS

The analysis results in five overarching themes. The themes include *providing opportunities for youth learning and growth*, *platforming youth*, *empowering youth*, *embracing youth leadership* and *promoting youth‐driven change*. The themes and their corresponding subthemes and supporting quotes can be viewed in Table 3.

**Table 3:** Summary of themes and corresponding quotes.

Themes	Quotes
1. Providing opportunities for youth learning and growth	
1.1 Deeper understanding of the MHA system	Mostly my interest was the opportunity to collaborate with other youth that have also had lived experience [with MHA] and then also see kind of what opportunities I can gain from learning other people's experiences and then sharing my own and then what can come out of that. (Participant 5)
	I'm also really interested in system navigation. And, the problems of system navigation specifically for youth. So I thought that this would be a really good opportunity to see what's being done in the FNP and what's being done elsewhere in the Greater Toronto Area and try to connect with folks who are interested in you know, the same things that I'm passionate about. (Participant 7)
1.2 Personal and professional growth	I'm trying to figure out where I want to be, if I want to be more behind the scenes or if I want to be patient facing. (Participant 7)
2. Platforming youth	
2.1 Elevating youth voice	My expectation is that they [FNP] might just be willing to listen to the input that we provide and also I want to see them be receptive. (Participant 4)
2.2 Meaningful youth engagement	There's for sure a difference between someone talking to you and then talking with someone on a collaborative format and kind of bouncing off opinions with each other. Rather than someone just talking at you for 20 minutes and you writing notes on something, I think it's more engaging for youth to feel more connected and not only that they're listening to something, but that they're contributing on an equal basis to everyone else. (Participant 5)
	Youth engagement means youth being able to be actively a part of creating a solution. (Participant 6)
3. Empowering youth	
3.1 Expectations of the organization	I think that was a really important thing that the youth engagement, the youth involvement was carried along throughout the entirety of that process. (Participant 4)
	I think the vibe of the ice breaker really helped. Like the story about the boomerangs, it made it feel less we're kids talking to adults, it made it seem like we're friends or co‐workers discussing fun things in our lives. (Participant 1)
3.2 Needed organizational support	I think there's a lot of new inclusive trainings that are out there, like some of the big ones are LGBQT2S+ trainings, other ones could be accessibility trainings and stuff like that, like universal design and things along that line. Because right now I feel like youth are at the forefront of a lot of these movements and are really strong advocates and having adults also being on the same page is really important, because then it shows that they're willing to prioritize youth in what they value first. Along that line, I think having trainings where youth voices are also heard is pretty important. (Participant 4)
3.3 Intentional adult interaction	If a youth said something that was unrealistic, instead of laughing and being like ‘oh that's a good idea but you're kind of way off.’ Being like, ‘that's a really good idea, now when I think about it we don't necessarily have that much funding so how can we do this in a smaller manner. What are your ideas?’ Instead of just, ‘ha‐ha that's funny or cute,’ and not really encouraging the next step. (Participant 8)
3.4 Organizational culture	I think being understanding and compassionate, but not being condescending [is important]. Adults can be very judgmental when you're someone clearly struggling. So I think [they could try] suspending judgement. Especially because, the talk around mental health is different than it was, even 10 years ago. So, I think it's important to suspend judgement and [to] be understanding. (Participant 6)
	There's a lot of times, where in cultural instances or in terms of sexuality and gender, there can be a lot of disconnect between younger people and older people. Just because of the way they were raised. So I think it's important to start really tackling that so that the services are suitable for all youth. (Participant 8)
	I feel like when I work with people who are definitely a couple of generations different from me, it feels very rigid and I don't have the opportunity to really speak in the way that I would normally speak just because we're so used to putting on this front to follow suit. (Participant 4)
4. Embracing youth leadership	
4.1 Comfortable environment	I felt like at least for a lot of the questions about personal experience during the interview it felt like a safe place to talk about it which I thought was very important, especially for mental health subjects. (Participant 1)
4.2 Collaborative practices	I'm a big believer in if you want to turn your camera off, turn the camera off. Or use the chat function, so I think that's just a little bit like, yes it's a little bit more inconvenient for everyone else, but at least you still are having the person who can't use these functions be a part of the process. And so that's why I like it when we do things other than just us talking on the screen. If there's other engagement opportunities that's a little bit more universal design I think that's always a benefit. (Participant 4)
4.3 Informed by youth experience	I know at least in group projects for school … I feel really bad when one person has a lot of work compared to everyone else. So I feel like one major thing is that everyone gets an equal amount of base work, and then should they want they can take on more things. (Participant 1)
4.4 Virtual tools for participation	I've kind of tried to navigate it [the MHA system] all over the world, and I've had this newcomer experience in Canada, and I've had to really try to find my own way through this really complicated mental health system. (Participant 4)
	One thing that I think could be pretty useful is a maybe like a Google document that we could use with everyone together. A lot of my work is currently based off of that, and having the accessibility for everyone. or like other team members to come in and put their own opinions or their thought process on what they're doing, and seeing what we're working on. I think it's a good idea to make it accessible, right? So that's one thing that I would like to see. (Participant 3)
5. Promoting youth‐driven change	
5.1 Youth impact	Especially after finding that help was really long, and just getting all that stuff sorted out was hard…that process is kind of daunting so I wanted to help out with it. (Participant 1)
	Having the youth's opinions and their thoughts is important for future decisions, they can't just let people decide what the world wants to make for them. (Participant 3)
5.2 Accountability	From this position you're seeing what happens with the navigators and what happens with that information, where does it go from there. And I found it really intriguing and helpful to understand what routes could be changed in the future, to be make it more beneficial and supportive to the individuals who are there. (Participant 3)
	If you want to create a space where youth feel like their contributions are valued, you need from the get go to establish that you genuinely care about their input. (Participant 4)
5.3 Outcomes	An opportunity for youth to have their say and have their voices be heard. I think that's really important, and so I really wanted to be a part of that. (Participant 4)

### 4.1 Providing opportunities for youth learning and growth

The youth shared their hopes for personal and professional learning through their participation in the YAC. This theme of providing opportunities for youth learning and growth included subthemes of *deeper understanding of MHA system* and *personal and professional growth*. This theme emphasized the importance of considering the benefits for youth joining a YAC in addition to the benefits organizations receive from establishing a YAC.

#### 4.1.1 Deeper understanding of MHA system

Youth discussed how their personal experience in the MHA system led them to be interested in how the system operates, and how it can be improved for other youth. They shared they wanted to learn more about MHA including navigation, peer support and community‐based organizations. Notably, the youth wanted to hear from peers about their experiences to inform their ideas and understanding of the MHA system. One youth shared,

I kind of just want to pick everyone's brain about what they've experienced [related to MHA] and what they've learned from it. Because I feel like what they've learned from, maybe I could apply to myself and then that could be applied to the broader community. (Participant 6)

In discussing their interest in learning more about the MHA system, youth identified hearing the experiences of peers seeking MHA care and the behind‐the‐scenes information from the service provider could help them to better determine where positive change could occur.

#### 4.1.2 Personal and professional growth

Youth advisors also recognized how this work could benefit themselves both personally and professionally. One youth shared, ‘I'm hoping to get some experience working in the mental health space and better understanding of how it works through talking to people who've also been in it. I can see myself in it in the future as well’ (Participant 5). Youth shared their excitement about learning about the different careers within the MHA field, with some youth having a clear idea about their career goals, while others wanted to use the opportunity to identify what kind of MHA career they were interested in.

On the other hand, some youth were not interested in pursuing a career in MHA but noted how the experience could provide transferrable skills such as facilitation, group work, advocacy and research skills. For example, one youth shared their goal as, ‘definitely learning more about the research aspect of things. Like how these studies are used to implement new structures in mental health service’ (Participant 8). Youth noted that these skills were valuable to helping them get ahead in school, ranging from adding to their resumes for graduate school applications to offering opportunities for volunteer hours for their secondary school graduation requirements.

### 4.2 Platforming youth

Youth advisors shared expectations that organizations should provide platforms for YACs to be heard and for their recommendations to be meaningfully actioned. This theme included subthemes of *elevating youth voice* and *meaningful youth engagement*. This theme highlights the importance of authentic youth engagement, where organizations go beyond creating a seat at the table for youth and take steps to ensure youth feedback will have a meaningful impact on service design, delivery and evaluation.

#### 4.2.1 Elevating youth voice

Youth advisors expressed that young people not only need MHA organizations to provide opportunities for them to share their ideas, opinions and feedback but that the organization must also be open to responding to it. Youth recognized the difference between being listened to and having their ideas impact programme direction. For these youth participants, being heard was just the first step, but having their voices result in meaningful change was their ultimate goal.

#### 4.2.2 Meaningful youth engagement

To achieve this meaningful impact, youth shared that they believed they should have robust involvement and influence over YAC activities. They indicated that when youth have this kind of involvement, they can structure the work in a way that continuously elevates youth voice. One youth shared an example of this robust involvement:

I really like how youth‐led everything is, I at first was a little bit hesitant. I thought it was going to be a little bit more passive. But then I think in the first meeting where we were talking about how if a youth is missing a meeting, how we were able to help catch them up, the idea of having another youth fill in that role. That was something that really stood out to me. (Participant 4)

In this example, the youth shared that it was important to them that the youth advisors be able to update their peers if they missed a meeting, so that the youth voice was not filtered through the adult lens of the organization liaison. Youth also discussed the importance of having a youth facilitate the YAC meetings. This allowed the youth to have control over their meetings and institute structures that helped their peers to feel engaged in the virtual setting, such as starting with an icebreaker. Involving youth at all stages of the project and giving them autonomy to choose how their participation was structured helped the youth to feel valued as they began their work on the YAC. Providing youth with a platform where the youth can have a meaningful impact and collaborate equally with adults in the space is critical for the authentic youth engagement required for YACs.

### 4.3 Empowering youth

Youth advisors shared their hopes and expectations that the organization would provide support to achieve their collective goal of uplifting youth voices within FNP. This theme of empowering youth included subthemes of *expectations of the organization*, *needed organizational support*, *intentional adult interaction* and *organizational culture*. This theme highlights that the organizational setting and adult support within it must empower youth to feel informed and valued, so that they are able to provide their feedback and embrace the importance of their role.

#### 4.3.1 Expectations of the organization

Youth reported different ways that they believed the organization would support them, including reflecting that they had the backing of FNP leadership and the organization liaison. Youth valued that FNP leadership demonstrated their support to the YAC through writing welcome letters, attending initial meetings to meet youth, making themselves available for one‐on‐one meetings with youth and encouraging youth to share their expectations in the group charter. Youth identified organizational support as playing a major factor in the sustainability of the project. YAC members requested that FNP leadership be available to support them in their project goals as needed, such as consulting on project direction and providing relevant contact information as it pertained to the project.

#### 4.3.2 Needed organizational support

Youth advisors reported that establishing relationships with FNP leadership and the organization liaison was critical to learn about organizational policies and practices to keep the YAC informed. One youth shared, ‘there has to be some kind of embedded staff support or otherwise it wouldn't work’ (Participant 2). Youth expected the organization liaison would provide necessary context in meeting discussion and allow for greater coordination between the YAC and FNP, which in turn would encourage organizational action on youth recommendations. Youth also shared the importance of administrative support, such as setting up the Zoom or emailing previous meeting notes, from the organization liaison to be able to make the most out of monthly meetings.

#### 4.3.3 Intentional adult interaction

Youth advisors also noted the importance of interaction with other adults within the organization. Youth reported that willingness on the adult's side to embrace youth ideas would be a critical part of the success of the work. Youth understood that the generational divide between youth and adults complicated the work ahead, but shared that they felt through open communication they could overcome any barriers to being seen as equal partners in the work. One youth shared, ‘I guess I expect a level of respect. Just because we're younger, doesn't mean our opinions should be any less valued’ (Participant 6). Youth also suggested that YAC interaction with adults on the team should be constructive and that adults should take a positive approach when working with youth. Youth described the role for adults within the organization on the YAC was to use their professional expertise to provide support and guidance for YAC activities. One youth shared,

Obviously, as much as I have lived experience, we [the YAC] don't have the professional experience or actual educational, clinical experiences and things like that. And so it's important for adults to have their professional expertise, who are also wanting to actually connect with youth and have an impact. So I think their role is very important, because they're the ones who can help facilitate and move things forward. So I think it's a collaborative effort. (Participant 4)

#### 4.3.4 Organizational culture

Furthermore, youth acknowledged systemic factors that contributed to how empowering the environment could be. Youth stressed the importance of diversity, equity, inclusion, accessibility and belonging on the YAC. Youth felt that it was critical for YACs to uplift the voices of youth who have historically been excluded from conversations regarding MHA care. One youth shared, ‘Having diversity is really important in all settings now, but especially the youth council. I think having that diversity of socioeconomic status, race, gender, sexuality … age. I think being able to have those perspectives is really beneficial’ (Participant 6).

Youth also shared that organizations preparing to work with youth should have staff with the capacity to engage youth with historically marginalized identities. The focus on diversity, equity, inclusion, accessibility and belonging felt important to youth as they noted the shifts over time in approaching and uplifting equity work. One youth shared, ‘I definitely think that there is a generational shift with the resistance that our generation holds against inequitable and unequal practice’ (Participant 7). Youth advisors were invested in creating an equitable YAC and expected that FNP would support their efforts. Youth recognized that especially in MHA care, where equity‐deserving communities have less access to care that is culturally sensitive and appropriate, it is critical to centre youth most marginalized by the system.

### 4.4 Embracing youth leadership

Youth advisors reflected on the importance of structuring the YAC in a way that was accessible and establishing collaborative practices to increase group cohesion. This theme of embracing youth leadership included subthemes of a *comfortable environment*, *collaborative practices*, *informed by youth experience* and *virtual tools for participation*. This theme reflects that youth advisors wanted to be able to lead the way to create an environment that would be open and honest.

#### 4.4.1 Comfortable environment

Youth stressed the importance of a comfortable environment for youth to be able to share authentic feedback. For example, one youth shared that having Youth Engagement Partners involved in the recruitment and interviews for the YAC showed that youth were valued at multiple facets of the initiative and helped them to feel more comfortable in the interviews.

Youth also wanted the meetings to feel informal. One youth shared, ‘I think, just, chatting more with the youth and making sure that it's not so formal, it doesn't have to feel like a meeting, like more of a conversation’ (Participant 5). Another youth acknowledged the difference between professionalism and authenticity, sharing that in adult workspaces it can be hard for youth to feel like they can be themselves. Instead, they wanted the YAC meetings to be spaces where youth could present themselves authentically and freely share what came to mind.

Youth advisors recommended prioritizing youth comfort in the space, through promoting honesty and respect from adults and each other. Youth attempted to cultivate this environment by highlighting the importance of positivity. One youth shared, ‘I always try and pick out the positive things. Which I think is a good trait, because it prevents stress and stuff from building up’ (Participant 1). Youth advisors also shared the importance of creating a culture of open mindedness and open communication on the YAC.

#### 4.4.2 Collaborative practices

Youth also reported important tactics for collaborating. Many youth had previous experience on group projects where they recognized the workload felt uneven, which contributed to their worries about how this group would work together. Youth shared concerns about group cohesion over the virtual environment but recommended strategies to help bridge the virtual divide and felt confident they could still establish positive relationships. Youth advisors recommended implementing tools such as creating a group charter and norms to help navigate any challenges that may arise. The YAC group norms included *actively listen to others*, *provide trigger warnings if possible* and *share the air—give everyone an opportunity to speak among others*. Setting up these agreements encouraged the collaborative environment by making expectations clear.

#### 4.4.3 Informed by youth experience

Youth noted their individual strengths, such as aspects of their identity and personality, as well as the lived and professional experience they brought into the work, were an asset for the YAC. Some youth highlighted that their lived experience in MHA services helped them to understand the unique needs of youth traversing MHA care. Another youth identified their professional skills as a benefit stating, ‘I've done a lot of research before at university, and I've been doing it at my current job. Figuring out creative and intriguing routes to go and look for what our project scope should be’ (Participant 3). Other youth identified being a team player, creativity and dedication to working hard as key strengths in working to promote positive change at FNP and the MHA system more broadly. Youth members hoped to utilize their individual and group strengths to achieve their goals.

#### 4.4.4 Virtual tools for participation

Finally, youth noted virtual tools for participation they felt were critical for engagement. For example, youth shared that using the online scheduling tool, Doodle, helped them to choose a standing time that worked for everyone for meetings. Youth also shared their appreciation of calendar invites and recap emails from the organization liaison to help them stay organized. Youth expected to be able to utilize virtual tools, such the Zoom chat, whiteboard functions and polls, and other online tools such as Google drive to achieve their individual goals to collaborate and remain engaged. They identified these tools as important to be able to move their work along for maximum impact.

### 4.5 Promoting youth‐driven change

Youth were motivated to join the YAC, and were most excited about, the opportunity to make a positive impact on the youth MHA system. This theme of promoting youth‐driven change included subthemes of *youth impact*, *accountability* and *outcomes*. Youth described the importance of feeling comfortable and confident in the organization to share their ideas with the goal of achieving positive change in the MHA system. This theme highlights the importance of organizational support to improve the MHA system through prioritizing youth feedback and ideas.

#### 4.5.1 Youth impact

Overall, youth shared the importance of youth impact through the YAC. One youth shared, ‘the objective of the YAC is specifically having a space in FNP to allow youth to have a contribution to how the organization moves forward to provide an outlet where youth are able to provide their input as to how an organization which focuses on families and youth will proceed’ (Participant 4). Youth reported the reason for their involvement was to provide youth feedback to the organization to best meet the needs of youth clients. They hoped to have a positive impact within FNP and to be able to share their ideas and the outcomes of the YAC throughout the sector to encourage a systemic shift towards meaningful inclusion and engagement of youth.

#### 4.5.2 Accountability

Youth advisors shared their goals to help other youth and guide FNP to be more youth‐friendly. Youth recognized that youth‐friendly services could reduce stigma for youth seeking care. One youth shared, ‘youth engagement means getting youth involved in their own care, and just getting youth more aware and more educated, breaking stigma and also just giving youth the space to be able to express their struggles’ (Participant 7). Many youths recognized that their task to complete a youth‐led project to increase youth engagement at FNP was a unique opportunity to have a meaningful positive impact. The youth felt dedicated to successfully completing the project. Some youth also discussed that being meaningfully embedded within FNP allowed them to identify areas for organizational improvement. Their expectations included that their partnership with FNP should be accountable to youth across the GTA and result in both the youth and organization working towards positive change.

#### 4.5.3 Outcomes

Youth shared they felt the YAC would be successful if youth were able to complete their chosen youth‐driven project. Youth also shared that they felt that youth involvement within youth MHA organizations more broadly helps to improve services through using firsthand experience to guide programme improvements. Youth hoped that their participation would lead to increased youth clients at FNP, increased youth ratings of FNP as youth‐friendly and more positive outcomes for youth seeking treatment such as improved mental wellness or access to culturally competent providers. In discussing their ideas for outcomes of the YAC, youth showed that they wanted tangible change.

## 5 DISCUSSION

This study explored the motivations, expectations, and goals of eight youths as they entered the work of a YAC within an MHA systems navigation organization. Youth were motivated by their lived experience in the MHA system to attempt to make it easier for other youth to access appropriate and timely care. Driven by this passion, youth had high expectations for their level of involvement, and a sense of how their strengths could contribute to positive change. Understanding youth motivations behind joining the YAC helps to clarify the intent of youth to create positive change for other youth in the MHA system. This finding is in line with previous work demonstrating youth are motivated by improving society for future generations.^43^

These youth expected FNP to move beyond surface‐level opportunities for impact, and to provide legitimate opportunities for youth voices to be heard and acted on. This is consistent with research in the field that emphasizes the importance of moving away from tokenization in youth engagement efforts.^18^, ^22^, ^25^, ^44^ Opportunities for youth to be empowered by organizations to make positive change are critical as previous research supports that youth must be empowered as partners in the work and be given platforms to draw on their lived experience to provide authentic feedback.^9^, ^15^ Youth have the right to be consulted on matters that impact them,^14^ and previous studies have demonstrated their input and feedback can, in turn, improve systems.^10^, ^27^

It is important that organizations create these opportunities for youth to drive positive change, but they must also pay careful attention to the youth's experience within the programme. To maximize the impact of youth engagement, organizations must be aware of the reasons youth are drawn to the work and help to support the kind of involvement they want to have. Results from this study support that youth are interested in taking part in leadership roles and utilizing their strengths to create collaborative and supportive advisory council environments. Youth advisors expected organizational support, respect from adults and for adult facilitators to be trained in working with equity‐deserving communities. Current research in youth engagement in healthcare has also supported the need for antiracist and equity‐focused programming.^44^, ^45^ Youth's expectations to be taken seriously and allowed to have meaningful impact adds to the current research, demonstrating authentic inclusion may also encourage youth to remain engaged.

The results of this study showcase the importance of structuring YACs with a strengths‐based view of youth and creating opportunities for youth to thrive. FNP used PYD to structure the YAC to underscore the importance of creating a mutually beneficial partnership. Previous research has demonstrated the value of youth–adult partnerships as a means to recognize the strengths of both youth and adults as critical in improving policy and practice.^9^, ^46^, ^47^ In this project, youth recommended that one way they would feel valued by adults as partners on the team would be for the organization liaison to report on the ways that the YAC was having an impact at FNP in each meeting to help youth to understand the impact they were having as a group and help keep the organization accountable to the YAC. Strategies such as these should be codeveloped in youth engagement efforts to promote healthy youth–adult partnerships. This study highlights that youth recognized their own strengths, experience and leadership capacity and expected that they would be provided space to utilize these skills. Youth are ready to be engaged as partners in this work, and organizations should move to see them in this way.

## 6 LIMITATIONS

Results from this study represent the views of eight YAC members at an MHA navigation organization in Toronto. It is possible that the focus of FNP on MHA navigation, rather than direct service delivery, impacted the way youth considered the scope of the project. It is also of note that measures were taken by the organization to approach the work democratically and to give youth power in the planning process. It is possible that these youth were primed to apply for this position because of this approach, and this could have skewed their motivations and expectations. However, insights from these eight youths can help to inform how other MHA organizations approach the planning and implementation of YACs to empower other youths to take on these leadership roles.

Furthermore, this study took place in the context of the COVID‐19 pandemic, meaning that YAC activities took place virtually. Thus, their comments regarding the environment often referred to virtual environments, and further exploration is needed regarding how these preferences may apply to in‐person settings.

Finally, while some of these youth had prior experience in youth engagement work, most of these youth were new to the experience. Future research should examine the perspectives of youth who have more experience with YACs to capture their reflections and recommendations for the practice.

## 7 CONCLUSION

This study explored the perceptions of youth entering their roles as youth advisors on a YAC for an MHA navigation service in Toronto, ON. Study findings contribute to a growing body of literature on youth engagement in MHA services by uncovering youth's motivations to join the council and their expectations coming into the work. The insights demonstrate youth are motivated to make positive changes for their peers and gain personal and professional skills. Youth expect their ideas and insights to be seriously considered and acted upon to improve the mental health system overall. A greater understanding of youth's motivations and expectations can help to inform organizational planning, implementation and evaluation processes for youth engagement through an advisory council structure.

## AUTHOR CONTRIBUTIONS

Adrienne Young conducted project administration, data collection, data analysis, and wrote the original draft of the manuscript. Roula Markoulakis provided supervision, provided methodology support, conducted data analysis, and reviewed and edited manuscript drafts. Roula Markoulakis, Sugy Kodeeswaran and Anthony Levitt contributed to conceptualization and funding acquisition. All authors read and approved the final manuscript.

## CONFLICT OF INTEREST STATEMENT

The authors declare no conflict of interest.

## Data Availability

The data that support the findings of this study are available from the corresponding author upon reasonable request due to privacy/ethical restrictions.
